# The costs of peer ethnic discrimination: Longitudinal associations with negative ethnic identity affect and identity coherence among ethnic minority adolescents

**DOI:** 10.1111/jora.70265

**Published:** 2026-09-28

**Authors:** Savaş Karataş, Jana Vietze, Chiara Ceccon, Ughetta Moscardino

**Affiliations:** ^1^ Department of Education and Pedagogy, Educational Psychology – Socialisation and Culture Research Group Martin Luther University Halle‐Wittenberg Halle (Saale) Germany; ^2^ College for Interdisciplinary Educational Research (CIDER) Frankfurt am Main Germany; ^3^ Department of Psychology, Education & Child Studies Erasmus University Rotterdam Rotterdam the Netherlands; ^4^ Department of Developmental Psychology and Socialisation University of Padova Padova Italy

**Keywords:** adolescence, discrimination, ethnic identity, identity coherence, longitudinal, negative ethnic identity affect, peer, youth

## Abstract

The present study examined longitudinal associations between perceived peer ethnic discrimination, negative ethnic identity affect, and global identity coherence among ethnic minority adolescents in Italy (*n* = 280; *M*_age_ = 14.29; 59.28% female; 76.42% second‐generation). Results indicated negative bidirectional associations between negative ethnic identity affect and global identity coherence. While the associations between peer discrimination and negative ethnic identity affect were bidirectional and positive, the links between peer discrimination and identity coherence were unidirectional and negative. Overall, the more negative feelings towards youth's own ethnic group membership were linked to lower identity coherence over time, and vice versa. The findings also reaffirmed the associations of peer discrimination with higher negative ethnic identity affect and lower global identity coherence, while reflecting that holding negative feelings about one's ethnic group may heighten the risk of being more sensitive and vulnerable to discrimination within peer context. Altogether, these findings offer a nuanced perspective on peer discrimination and its interplay with youth identity development in Italy.

## INTRODUCTION

*“*Who am I?” is the core identity question that young people strive to find a meaningful answer to throughout their lives. During adolescence, integrating one's identity across multiple life domains forms one of the central developmental tasks (Erikson, 1968). However, forming a coherent sense of *overall* self (i.e., global identity coherence) might be highly challenging for those navigating across two or more cultural reference points, which is often the case for ethnic minority youth (Suárez‐Orozco et al., 2018; see also Crocetti et al., 2011, 2024). In other words, for youth who were themselves born outside of the destination country or with at least one parent who was born outside of the destination country (i.e., ethnic minority youth; European Commission, 2020), this involves such complex tasks as integrating identities that are derived from ethnic group membership (i.e., ethnic identity) next to developing a clear, coherent sense of self, both of which are inextricably intertwined (e.g., Mastrotheodoros et al., 2021; Meca et al., 2017).

Feeling positively about their ethnic group membership (i.e., positive ethnic identity affect) may contribute to forming a coherent sense of overall self among youth and vice versa (Meca et al., 2017). Along this line of thought, negative feelings towards one's ethnic group membership (i.e., negative ethnic identity affect) may be adversely consequential for the formation of coherent identities and be strengthened by difficulties achieving such coherence. For the development of both ethnic and coherent identities, however, perceiving differential and detrimental treatment based on youth's own ethnic group membership (i.e., ethnic discrimination) may pose a substantial risk.

Based largely on studies conducted in the United States, research to date has shown a relatively consistent pattern of negative associations between ethnic discrimination and identity coherence (e.g., Meca, Webb, et al., 2022). In contrast, findings on its role in ethnic identity formation remain rather mixed. Ethnic discrimination may sometimes strengthen ethnic identity (e.g., Umaña‐Taylor & Guimond, 2010), yet ethnic identity can also influence how discrimination is experienced (e.g., Sellers & Shelton, 2003). Although more recent U.S.‐based studies pointed out potential bidirectionality (e.g., Cheon & Yip, 2019; Meca et al., 2020), some also underscored that discrimination might nonetheless have harmful effects (Zeiders et al., 2019), especially when the perpetrators are members of microsystems, such as the peer group (e.g., Del Toro et al., 2021; Stein et al., 2023). Hence, to better understand the interplay of ethnic identity and discrimination, it is of utmost importance to adopt a domain‐specific approach to discrimination.

Given that peers become an increasingly central life domain during adolescence (Brown & Larson, 2009; Karataş, 2025), the impacts of ethnic discrimination on increasing negative feelings towards one's own ethnic identity and diminishing overall identity coherence might be heightened when youth experience ethnic discrimination within peer interactions (for ethnic identity, see Del Toro et al., 2021). Along this line of thought, it is of utmost importance to revisit the timeless “Who am I?” question with a slight reframing that speaks to the lived experiences of ethnic minority adolescents: *Who am I, especially when my peers treat me as “different” because of my ethnic and cultural background?* To address this, the current three‐wave study adopts a domain‐specific approach to discrimination in order to better understand how perceived peer ethnic discrimination is longitudinally associated with negative ethnic identity affect and global identity coherence among ethnic minority adolescents in Italy.

### Building a coherent self: Interrelations with ethnic identity in adolescence

Building a coherent sense of self is a critical developmental task over the course of adolescence (Erikson, 1968). In increasingly diverse societies, addressing this fundamental task might be more intensive for ethnic minority youth (Crocetti et al., 2011, 2024), as they are also required to fulfill additional acculturative tasks, such as establishing ethnic identity or developing a clear understanding of their belongingness to a particular ethnic group (for a review, see Suárez‐Orozco et al., 2018). The main tenets of social identity perspectives (Tajfel & Turner, 1986) suggest that youth learn who they are as a result of evaluative processes in which they compare themselves to others. Accordingly, ethnic minority youth might tend to favorably regard their membership in a particular ethnic group (i.e., ingroup) and differentiate themselves from the others (i.e., outgroup). As such, their ethnic identity may shape how they see themselves and can also give them a sense of direction when they explore, commit to, or reconsider shared traditions, values, knowledge, and history through interactions with other people from their own and other ethnic groups. In other words, adolescents' self‐concepts might be informed by their socially constructed context‐dependent ethnic identities (Umaña‐Taylor, 2024).

Although ethnic minority adolescents may need more time to form a coherent sense of overall self, possibly because it takes them longer to explore and reconsider their personal identities (Crocetti et al., 2011), identity coherence may also anchor ethnic minority youth during cultural exchange (or transition) and adaptation (Schwartz et al., 2006). Indeed, ethnic minority youth resilience models (Motti‐Stefanidi et al., 2021; Suárez‐Orozco et al., 2018) have argued that ethnic identity formation (as acculturative processes; see Schwartz et al., 2010) is likely accelerated or impeded if they match or mismatch with core developmental processes, including forming a coherent sense of self across life domains. Thus, forming a coherent sense of overall self and ethnic identity formation should be considered in tandem.

Ethnic identity is a multidimensional construct that includes both the *process* and *content* dimensions of ethnic and cultural group membership (Umaña‐Taylor et al., 2014). Building predominantly upon Erikson's (1968) identity conceptualization and Marcia's (1966) Ego‐Identity Status Model, ethnic identity is operationalized as a developmental process (Phinney, 1989) in which young people seek information about their ethnic and cultural group (i.e., exploration) and develop a clear understanding of the meaning and sense of this group membership (i.e., commitment). Complementing this developmental perspective and drawing on the main tenets of Social Identity Theory (Tajfel & Turner, 1986), Phinney (1990) further emphasized ethnic identity affirmation as also representing the dimension of ethnic identity content (see also Umaña‐Taylor et al., 2004). Ethnic identity affirmation pertains to broader attitudes and behaviors grounded on the “positive” feelings towards one's ethnic group (i.e., positive ethnic identity affect; Umaña‐Taylor et al., 2004, 2014). Although they differ in terms of their conceptual origin and focus, ethnic identity centrality and private regard respectively capture the significance and meaning of belonging to an ethnic group (Sellers et al., 1998), and likewise reflect positive ethnic identity affect or its importance (Meca, Gonzales‐Backen, et al., 2022; Rivas‐Drake et al., 2014; Umaña‐Taylor et al., 2014).

Ethnic identity affect involves both positive and negative affect. However, the dearth of work with ethnic minority youth has predominantly focused on the positive ethnic identity affect and has demonstrated its associations with a broad array of positive outcomes, including lower depressive symptoms and higher social functioning, self‐esteem, well‐being, and academic adjustment (for a meta‐analysis, see Rivas‐Drake et al., 2014). In most studies, positive ethnic identity affect has been assessed using reverse‐coded negatively worded items, which may reflect “a lack of overtly negative feelings” rather than stronger positive affect (Meca, Gonzales‐Backen, et al., 2022, p. 494). Notably, these negatively worded items exhibited stronger factor loadings on negative ethnic identity affect (Meca, Gonzales‐Backen, et al., 2022) that might be linked to adverse self‐evaluation and maladjustment (for a review, see Umaña‐Taylor, 2024). In a recent U.S. study, negative ethnic identity affect was indeed positively associated with depression and anxiety and negatively linked to psychological well‐being, and its associations with depression and anxiety were stronger than those of general ethnic identity affirmation (Meca, Gonzales‐Backen, et al., 2022). Accordingly, distinguishing negative ethnic identity affect is essential for accurately reflecting ethnic identity content. To the best of our knowledge, no studies have thus far examined the interplay of ethnic identity and identity coherence in light of the recently recommended conceptualization of negative ethnic identity affect.

Whereas their focus was primarily on ethnic identity processes rather than positive or negative affect, only a few studies have examined the longitudinal interplay between personal and ethnic identities. In the European context, Mastrotheodoros et al. (2021) found a negative longitudinal association between exploration of ethnic and personal identities, alongside consistent positive within‐time correlations between the two processes. In their cross‐sectional study including alternative model testing, Safa et al. (2024) examined ethnic identity achievement (i.e., higher exploration and resolution) and affirmation separately among ethnic minority youth in the United States and revealed bidirectional associations of global identity coherence with ethnic identity achievement, but not with affirmation. In another study, where ethnic identity affirmation and commitment were combined into a single construct, Meca et al. (2017) found a bidirectional association between ethnic identity and identity coherence among Hispanic adolescents in the United States. That is, the more ethnic identity affirmation/commitment was linked over time to increased identity coherence and vice versa. Considering this mutually reinforcing relationship between ethnic identity affirmation/commitment (reflecting positive affect) and identity coherence, negative bidirectional associations between negative identity affect and identity coherence are likely. This interplay nonetheless awaits empirical examination, particularly against the backdrop of ethnic discrimination, as the fulfillment of both identity tasks unfolds within societal contexts (Branje, 2022; Umaña‐Taylor et al., 2014) that often involve discrimination.

### Adopting a domain‐specific approach to discrimination: Peer discrimination and its associations with ethnic identity and identity coherence

Identity development does not happen in a social vacuum but in a complex interplay of global forces, political and social contexts, and especially microsystems, including the family, school, and peers (Branje, 2022; Umaña‐Taylor et al., 2014). Within these microsystems, the development of a coherent sense of self can be supported but also threatened, for instance, by repeated experiences of ethnic discrimination. In fact, studies on Cultural Stress Theory (Salas‐Wright & Schwartz, 2019; Schwartz et al., 2015) identified perceived discrimination as one of the major stressors, together with a negative context of reception and bicultural stress. Because these stressors, including perceived discrimination, consistently convey messages of exclusion and rejection, they might hinder the development of a coherent sense of self (Meca, Webb, et al., 2022) or at least increase its instability over time (i.e., fluctuations; Schwartz et al., 2024).

Although personal and ethnic identities reinforce each other (Meca et al., 2017), or at least co‐develop (Mastrotheodoros et al., 2021), during adolescence, and although the adverse consequences of ethnic discrimination for identity coherence are well established (e.g., Meca, Webb, et al., 2022), the interplay between discrimination and ethnic identity remains rather complex. Herein, two competing, yet not mutually exclusive, conceptual models guide the understanding. On the one hand, the Rejection‐Identification Model (RIM, Branscombe et al., 1999) asserts that individuals who experience discrimination may become more aware of their membership in a marginalized group, and therefore identify more highly with their ethnic group as a result (for a review, see also Yip, 2018). Within this framework, the heightened identification is expected to contribute to well‐being, and has been shown to buffer against future (chronic) experiences of rejection and protect against delinquent behavior (e.g., Mazzoni et al., 2020). Despite its strong theoretical foundations, RIM has been replicated for ethnic identity commitment among college students with low national orientation (Fuller‐Rowell et al., 2013) and, more importantly, for positive ethnic identity affect (i.e., affirmation) among male adolescents (Umaña‐Taylor & Guimond, 2010).

On the other hand, the Identification‐Attribution Model (IAM, Gonzales‐Backen et al., 2018) offers an alternative perspective by suggesting that adolescents might become more aware of the instances of discrimination directed at their ethnic group when they develop ethnic identity. This model argues that higher ethnic identity exploration may increase adolescents' awareness of the history and reality of ethnic stratification and consequently contribute to stronger discrimination perception. Similarly, when adolescents endorse stronger ethnic identity commitment, they may also be more likely to attribute negative social experiences to discrimination because such social experiences conflict with their positive feelings towards, and a more fine‐grained understanding of, ethnic identity. Although their study sustained these assumptions only for ethnic identity exploration (Gonzales‐Backen et al., 2018), another study focusing on ethnic identity centrality among young adults in the United States (Sellers & Shelton, 2003) has indicated that perceived discrimination tends to be higher among individuals who place greater importance on their ethnic identity. As such, ethnic identity may influence perceptions of ethnic discrimination.

Recent longitudinal studies have supported the assumptions of both models. In their study with first‐ and second‐generation ethnic minority youth in the United States, Meca et al. (2020) demonstrated that ethnic identity commitment was positively associated over time with discrimination, while discrimination was positively linked to ethnic identity exploration at a later time point. Another study conducted with Asian and Latinx adolescents in the United States further underscored the similar bidirectional pattern between process dimensions of ethnic identity and discrimination among Asian adolescents. Importantly, among Latinx adolescents, private regard was positively linked to decreased discrimination at a later time point. Heightened discrimination was in turn associated with increased ethnic identity centrality (Cheon & Yip, 2019). Whereas these findings suggest that ethnic discrimination might be differently linked to process and content dimensions, interpreting them in light of the theoretical underpinnings of both RIM and IAM implies potential bidirectional associations between negative ethnic identity affect and discrimination.

However, the valence of this potential bidirectional pattern can hardly be anticipated because negative links between discrimination and ethnic identity have also been demonstrated, especially with positive identity affect (i.e., affirmation; Zeiders et al., 2019; private regard; Cheon & Yip, 2019). Importantly, the detrimental effects of discrimination may be heightened when the perpetrators are peers (Del Toro et al., 2021). Peers constitute a salient acculturative context (for a review, see Karataş, 2025) in which adolescents learn about and maintain cultural practices (e.g., Karataş, Crocetti, et al., 2023; Vietze et al., 2019; Wang & Benner, 2016), particularly because youth spend increasing amounts of time with peers during adolescence (Brown & Larson, 2009). A recent longitudinal study with early adolescents in the United States has indeed revealed that perceived peer discrimination predicted lower positive ethnic identity affect (i.e., private regard), and these effects were significant only when the perpetrators of discrimination were peers (Del Toro et al., 2021). Moreover, another longitudinal study of Mexican‐origin youth in the United States also showed that peer discrimination decreased the positive ethnic identity of youth over time (Stein et al., 2023). These findings not only diverge from the expected valence driven by RIM and IAM, but also underpin the domain‐specific nuances of discrimination during adolescence.

Overall, if peer discrimination has adverse consequences on ethnic identity, including exacerbating negative ethnic identity affect, its effects may inevitably extend to identity coherence due to the mutually reinforcing nature of both identities (e.g., Meca et al., 2017). However, extant studies have primarily focused on ethnic discrimination without applying a domain‐specific lens (for the exceptions, see Del Toro et al., 2021; Stein et al., 2023) and have addressed its interplay with either ethnic identity (e.g., Zeiders et al., 2019) or coherent identity development (e.g., Schwartz et al., 2024). Of these studies focusing on peer discrimination, most captured either ethnic identity processes or positive ethnic identity affect while leaving negative ethnic identity affect unexamined. Therefore, the current study adopts a domain‐specific approach to discrimination and disentangles whether and how peer ethnic discrimination is longitudinally related to negative ethnic identity affect and identity coherence among ethnic minority youth in Italy. In doing so, this study may contribute to a broader understanding of this interplay beyond the United States, where most available knowledge is produced.

### Discrimination and identity dynamics in Italy

As one of the main European destinations for immigrants, Italy is at the forefront in the process of managing ethnic and cultural diversity. In 2023, over 870,000 students without Italian citizenship were enrolled in schools. This represents about 10% of the total student population, with about two‐thirds of them born in Italy (ISTAT, 2024). Although national policies formally promote intercultural inclusion, emphasizing Italian language acquisition and civic participation (Ministero dell'Istruzione, 2022), implementation remains uneven. For example, many educational settings in practice continue to reflect orientations that privilege conformity to mainstream cultural norms over reciprocal adaptation or intercultural dialogue (Kosic et al., 2005). However, a few structured opportunities for intercultural learning have also been introduced (see, e.g., Ceccon et al., 2023).

Despite these limited opportunities, a broader assimilationist tendency in Italian public discourse, where diversity is mostly treated as a temporary condition to be managed rather than as a structural feature of society (Caritas, 2025) may restrict the recognition of heritage languages and identities and even exacerbate the stigmatization of individuals with diverse ethnic and cultural backgrounds through exclusive labels such as “foreigners” or “immigrants” (Levy, 2015; Moffitt et al., 2020). The endorsement of these labels may stem from the conflation of ethnic and national identities (i.e., ethnic nationalism; Brubaker, 2009). This conflation becomes inherently exclusionary because national (e.g., Italian) identity is reserved for members of the majority group, whereas individuals from minority backgrounds, although born and/or raised in the country, continue to be described as “immigrants” or “foreigners”. Apart from their inherent nature in increasing social divides, these exclusive labels, not only in Italy but also across larger Europe (see Moffitt et al., 2018), may lead to an overlap between immigrant and ethnic minority status, at least in the public discourse and perception.

Implicit pressures towards assimilation and limited institutional acknowledgment of diversity within such a normative broader climate of Italy may sustain both subtle hierarchies of belonging and everyday forms of ethnic discrimination, even in peer contexts. In addition, public discourse and media narratives further reinforce these expectations, portraying immigration as a challenge to social cohesion or national identity (Rubaltelli et al., 2020). Given the relatively assimilationist dynamics of the Italian context, more systematic evidence is needed to clarify the role of peer discrimination in driving ethnic and personal identities among ethnic minority youth. This way, it could be possible to develop a culturally nuanced understanding of the development of cohesive ethnic and personal identities in adolescence.

### The current study

Ethnic identity and forming a coherent overall sense of self are closely intertwined due to their promotive and mutually reinforcing nature during adolescence. However, the valence of their interplay might be opposite when negative (versus positive) ethnic identity affect is considered. In this vein, it is plausible to expect the bidirectional and negative associations between negative ethnic identity affect and global identity coherence among youth. Given that both identities unfold within broader societal contexts, it is crucial to explore this interplay against the backdrop of peer ethnic discrimination. While discrimination may pose a high risk to identity coherence, its associations with negative ethnic identity affect are still unclear, as it can be unidirectional or bidirectional and positive or negative. Accordingly, the main purpose of this study remains exploratory and examines how peer ethnic discrimination is related over time to negative ethnic identity affect and identity coherence among ethnic minority youth in Italy. This way, it would be possible to add to the canon of European longitudinal studies that inquire into the directionality of associations, whether effects are unidirectional or bidirectional, and their valence, whether the relations are positive or negative.

## METHODS

### Participants

Participants were recruited in a city in Northeastern Italy, an area with a high proportion of legally residing citizens of immigrant origin (ISTAT, 2024), specifically in three public upper secondary schools involved in the implementation of the Identity Project intervention (see Procedure section). For the purposes of the current study, only data from ethnic minority participants were selected from the larger sample (*N* = 698). Hence, the final sample included 280 adolescents (*M*_*age*_ = 14.29 years, *SD* = .86, range = 13–18). Of these, 59.28% self‐identified as female, 38.92% as male, 0.71% as non‐binary, and the remaining participants either specified another gender or did not prefer to disclose their gender. The majority of the participants, 76.42%, were born in Italy (i.e., second‐generation), whereas first‐generation youth had lived in Italy for 8.16 years on average (*SD* = 4.20, range = 3–14 years). Overall, participants reported 53 different countries of origin (also including parents' birth countries), the largest ones being Romania (17.14%), Morocco (12.68%), Moldova (7.50%), China (7.32%), and Albania (6.96%) based on families' backgrounds. Detailed information on participants and their parents' national origin is provided in the Supporting Information (see Table S1).

### Measures

#### Peer discrimination

Peer discrimination was measured using the discrimination subscale of the Peer Ethnic‐Racial Processes Measure (Wang, 2021). The original subscale consists of 5 items capturing unfair experiences in mild forms. However, due to lower standardized factor loadings, one item (“Felt that other kids were afraid of me because of my culture of origin”) was excluded, and 4 items were used in the present study (for more information, see Supporting Information). Participants were asked to indicate whether the behaviors described in each item had happened to them in the last 7 days on a 3‐point Likert‐type rating scale (1 = *never*, 2 = *sometimes*, or 3 = *often*). Sample item includes: “Been treated unfairly by other kids because of your culture of origin”.

#### Negative ethnic identity affect

The negative affect subscale of the Ethnic Identity Scale (EIS; Umaña‐Taylor et al., 2004) was used to assess the extent to which participants feel negatively about their ethnic (i.e., cultural) group membership. The subscale comprises 6 items rated on a 4‐point Likert‐type rating scale (1 = *does not describe me at all*; 4 = *describes me very well*). Initially, participants were asked to indicate their heritage culture (e.g., Italian, Romanian, Moroccan, Chinese) and think about that particular ethnic group when answering the questions. A sample item is: “I feel negatively about my ethnic identity”.

#### Global identity coherence

The identity synthesis subscale of the Erikson Psychosocial Stage Inventory (EPSI; Rosenthal et al., 1981) was used to assess global identity coherence. The original subscale is composed of 6 items. However, due to lower standardized factor loadings, one item (“I have a strong sense of what it means to me to identify with my gender [male, female, non‐binary, …]”) was excluded in this study (see Supporting Information). Each item was rated on a 5‐point Likert‐type rating scale (1 = *strongly disagree*; 5 = *strongly agree*).

#### Covariates

Participants' age and self‐identified gender (0 = *male*, 1 = *female*) were treated as covariates.

### Procedure

Participants in this study were drawn from the second edition of the Identity Project Intervention implemented in the school year 2022–2023. After obtaining approval for the study from the Ethics Committee of the School of Psychology at the University of Padova (protocol n. 3871), the research team contacted the teachers and principals of the schools, with whom collaborations had been established during the Identity Project implementation conducted in the previous school year (see Ceccon et al., 2023). The Identity Project is a school‐based curriculum originally designed in the United States to promote ethnic‐racial identity development (e.g., Umaña‐Taylor, Douglass, et al., 2018; Umaña‐Taylor, Kornienko, et al., 2018). The intervention was subsequently culturally adapted and implemented in several European countries (see Juang et al., 2023), including Italy (Ceccon et al., 2023). Throughout 8 weekly sessions carried out during school hours, participants are engaged in activities and reflections concerning personal and social identities, stereotypes and discrimination, meaningful cultural symbols, and family heritage.

Before conducting the study, written informed consent forms were distributed to the students and asked to be returned signed by parents and adolescents themselves to confirm participation in the research. The variables of interest were measured 1 week prior to the intervention (T1, October 2022), 1 week after the intervention, i.e., 9 weeks after baseline (T2, December 2022), and 13 weeks after baseline (T3, January 2023). Participants completed the survey via an online platform (i.e., Qualtrics) and could choose among Italian, Arabic, Brazilian Portuguese, Chinese, English, and French languages. These specific versions were made available based on participants' previous requests and prepared using standard translation‐back translation techniques.

### Strategy of analysis

Preliminary analyses (i.e., missing value analyses, descriptive statistics, bivariate correlations, and intraclass correlations) were performed using IBM SPSS version 28. The rest of the analyses were completed in M*plus* 8.6 (Muthén & Muthén, 1998–2017) with a maximum likelihood estimator with robust standard errors (Satorra & Bentler, 2001). Model fit was evaluated by considering the following criteria: Comparative Fit Index (CFI) with values higher than .90 and .95 suggesting an acceptable and excellent fit, respectively; and the Standardized Root Mean Square Residual (SRMR) and the Root Mean Square Error of Approximation (RMSEA) with values below .08 and .05, respectively, indicating acceptable and excellent fit (Byrne, 2012). Additionally, a 90% Confidence Interval (CI) for the RMSEA is considered acceptable if its upper bound is lower than .10 (Chen et al., 2008). While comparing the nested models, the Satorra‐Bentler scaled chi‐square difference test (Satorra & Bentler, 2001) and changes in parameter estimates (i.e., ΔCFI and ΔRMSEA; Cheung & Rensvold, 2002) were evaluated. Invariance was established if at least two of the following criteria were met: non‐significant ${\Delta \chi }_{\mathrm{SB}}^{2}$ (Satorra & Bentler, 2001), ΔCFI < −.010, and ΔRMSEA < .015 (Chen, 2007).

Prior to conducting the main analyses, longitudinal measurement invariance for each study variable was tested. Detailed information about this procedure is available in Supporting Information. Afterward, to disentangle the longitudinal interplay of perceived peer discrimination, negative ethnic identity affect, and identity coherence, a cross‐lagged panel model with manifest variables was estimated. In this model, (a) cross‐lagged paths were estimated by controlling for (b) stability paths across successive time points (T1 → T2 and T2 → T3), (c) within‐time correlations (at T1, and correlated changes at T2 and T3), and (d) covariate effects (T1 → T2 and T1 → T3). To establish the most parsimonious model while testing for time invariance of effects, five alternative models were estimated, where stability paths (M2), cross‐lagged effects (M3), correlated changes (M4), and covariates' effects (M5) were fixed to be equal across time points. Finally, two sets of multigroup analyses were conducted to test whether model results remained stable across youth's participation in the Identity Project Intervention (i.e., intervention and control groups) and generational statuses (i.e., first‐generation and second‐generation ethnic minority adolescents). To this end, a constrained model in which all model parameters were set to be equal across groups was compared with an unconstrained model where all model parameters could vary across groups.

### Researcher reflexivity statement

The authors of the current research hold multiple ethnic and cultural identities and social positions, representing non‐European (i.e., Türkiye), Western European (i.e., Germany), and Southwestern European (i.e., Italy) contexts. All scholars have either experienced migration or belonged to an ethnic minority during childhood, adolescence, or (emerging) adulthood. Some have been identified as ethnic minorities with extensive experiences of racialization in their destination countries and have been engaged in the development of a multicultural identity throughout adolescence or (emerging) adulthood. These facets of the authors' identities inevitably shape the current research questions and enrich the interpretation of findings.

## RESULTS

### Preliminary results

Means, standard deviations, and bivariate correlations among study variables are presented in Table 1. Because of the nested structure of the data (i.e., students nested in classrooms), intraclass correlations were calculated. The findings revealed low between‐group variance with values ranging from 0.018 to 0.024 (average = 0.022) at T1, from 0.008 to 0.053 (average = 0.037) at T2, and from 0.003 to 0.032 (average = 0.014) at T3.

**TABLE 1:** Descriptive statistics, Cronbach's alpha coefficients, and bivariate correlations among study variables.

	*M*	*SD*	*α*	1	2	3	4	5	6	7	8
Peer discrimination T1	1.609	.517	.777	**−**							
2 Negative ethnic identity affect T1	1.360	.509	.812	.122*	**−**						
3 Identity coherence T1	3.240	.855	.806	−.222***	−.199**	**−**					
4 Peer discrimination T2	1.613	.497	.768	.477***	.145*	−.066	**−**				
5 Negative ethnic identity affect T2	1.372	.532	.852	.143*	.582***	−.216**	.138*	**−**			
6 Identity coherence T2	3.189	.844	.810	−.180**	−.217**	.753***	−.068	−.163*	**−**		
7 Peer discrimination T3	1.606	.516	.805	.465***	.123	−.151*	.587***	.166*	−.131	**−**	
8 Negative ethnic identity affect T3	1.421	.555	.836	.143*	.471***	−.231***	.235***	.635***	−.197**	.261***	**−**
9 Identity coherence T3	3.243	.860	.816	−.230***	−.268***	.620***	−.141*	−.184**	.688***	−.189**	−.290***

Abbreviation: T, time.

* *p* < .05.

** *p* < .01.

*** *p* < .001.

Missing data ranged from 4.3% to 16.1% across three time points. At T1, rates for peer discrimination, negative ethnic identity affect, and identity coherence were 6.4%, 4.3%, and 6.1%, respectively; corresponding rates at T2 were 16.1%, 15.0%, and 15.4%, and at T3 were 9.3%, 7.9%, and 8.6%. For the covariates, missing data rates were 1.8% for age and 0.7% for gender. The results of Little's (1988) missing completely at random (MCAR) test yielded a normed *χ* ^2^ (*χ* ^2^/*df*) of 1.169, indicating that data were likely missing at random.

Detailed information about the procedure and the results of the longitudinal measurement invariance testing is presented in the Supporting Information (Tables S2, S3, and S4). The results of the longitudinal measurement invariance indicated that at least partial metric invariance could be achieved for all study measures (Table S2). However, standardized factor loadings of some items of the peer discrimination and identity coherence measures were relatively low (Table S3). Therefore, another set of longitudinal measurement invariance tests was conducted by excluding those poorly loaded items (Table S4). As a result, full metric invariance was achieved for the measures of peer discrimination and identity coherence.

### Main results

As shown in Table 2, the comparisons of the nested models indicated that time‐invariance could be established for (a) stability paths (T1 → T2 and T2 → T3), (b) cross‐lagged effects (T1 → T2 and T2 → T3), (c) correlated changes (at T2 and T3), and (d) covariates' effects (T1 → T2 and T1 → T3). Thus, the model that includes all these time constraints (M5) was considered the most parsimonious final model. The complete model results are reported in Table 3.

**TABLE 2:** Cross‐lagged models including the time‐invariant effects of the covariates: model fit indices and model comparisons.

Models	Model fit indices						Model comparison				
	*χ* ^2^	*df*	CFI	SRMR	RMSEA [90% CI]	Models	${\Delta \chi }_{\mathrm{SB}}^{2}$	Δ*df*	*p*	ΔCFI	ΔRMSEA
M1: Baseline model	46.225	15	.949	.056	.087 [.059, .117]						
M2: Model with time‐invariance of stability paths	48.741	18	.949	.060	.079 [.053, .106]	M2‐M1	2.592	3	.459	.000	−.008
M3: Model with time‐invariance of stability paths and cross‐lagged paths	51.052	24	.955	.064	.064 [.040, .089]	M3‐M2	3.123	6	.793	.006	−.015
M4: Model with time‐invariance of stability paths, cross‐lagged paths, and T2– T3 correlations	54.982	27	.954	.066	.062 [.038, .085]	M4‐M3	4.310	3	.230	−.001	−.002
M5: Model with time‐invariance of stability paths, cross‐lagged paths, and T2– T3 correlations, and covariate effects	58.674	33	.958	.065	.053 [.030, .075]	M5‐M4	2.629	6	.854	.004	−.009

Abbreviations: CFI, Comparative Fit Index; *df*, degrees of freedom; RMSEA [90% CI], Root Mean Square Error of Approximation and 90% Confidence Interval; SRMR, Standardized Root Mean Square Residual; TLI, Tucker‐Lewis Index; Δ, Change in the parameter; $\chi_{\mathrm{SB}}^{2}$, Satorra‐Bentler scaled chi‐square.

**TABLE 3:** Standardized results of the cross‐lagged model.

**Stability paths**	**T1 → T2**	**T2 → T3**	
Peer discrimination	.525***	.538***	
Negative ethnic identity affect	.583***	.631***	
Identity coherence	.714***	.677***	
**Cross‐lagged paths**	**T1** → **T2**	**T2** → **T3**	
Peer discrimination → Negative ethnic identity affect	.140***	.138***	
Peer discrimination → Identity coherence	−.065*	−.063*	
Negative ethnic identity affect → Peer discrimination	.118**	.134**	
Negative ethnic identity affect → Identity coherence	−.072*	−.077*	
Identity coherence → Peer discrimination	.006	.006	
Identity coherence → Negative ethnic identity affect	−.075*	−.072*	
**Covariate effects** ^a^	**T1** → **T2**	**T1** → **T3**	
Age → Peer discrimination	−.005	−.005	
Age → Negative ethnic identity affect	−.024	−.024	
Age → Identity coherence	−.011	−.010	
Gender → Peer discrimination	.004	.004	
Gender → Negative ethnic identity affect	−.071*	−.070*	
Gender → Identity coherence	−.056	−.054	
**Within‐time correlations**	**T1**	**T2**	**T3**
Peer discrimination ↔ Negative ethnic identity affect	.126	.030	.033
Peer discrimination ↔ Identity coherence	−.224***	−.024	−.023
Negative ethnic identity affect ↔ Identity coherence	−.186**	−.088	−.086

Abbreviation: T, time.

^a^ Five participants had missing age and/or gender data, and three identified as non‐binary, another gender, or undisclosed gender. Therefore, final analyses were completed with the sample of 272 participants.

* *p* < .05.

** *p* < .01.

*** *p* < .001.

As displayed in Figure 1, the cross‐lagged panel model results revealed the expected negative bidirectional links between negative ethnic identity affect and identity coherence, indicating that higher negative ethnic identity affect was associated over time with relatively lower identity coherence (*β* = −.072, *p* < .05 between T1 and T2; *β* = −.077, *p* < .05 between T2 and T3) and vice versa (*β* = −.075, *p* < .05 between T1 and T2; *β* = −.072, *p* < .05 between T2 and T3). Importantly, the positive bidirectional longitudinal associations between perceived discrimination and negative ethnic identity affect were also captured. That is, among ethnic minority adolescents, higher perceived peer discrimination was related to relatively higher negative ethnic identity affect at a later time point (*β* = .140, *p* < .001 between T1 and T2; *β* = .138, *p* < .001 between T2 and T3), and vice versa (*β* = .118, *p* < .01 between T1 and T2; *β* = .134, *p* < .01 between T2 and T3). Lastly, the higher perceived peer discrimination was related to relatively lower global identity coherence over time (*β* = −.065, *p* < .05 between T1 and T2; *β* = −.063, *p* < .05 between T2 and T3).

**FIGURE 1:**
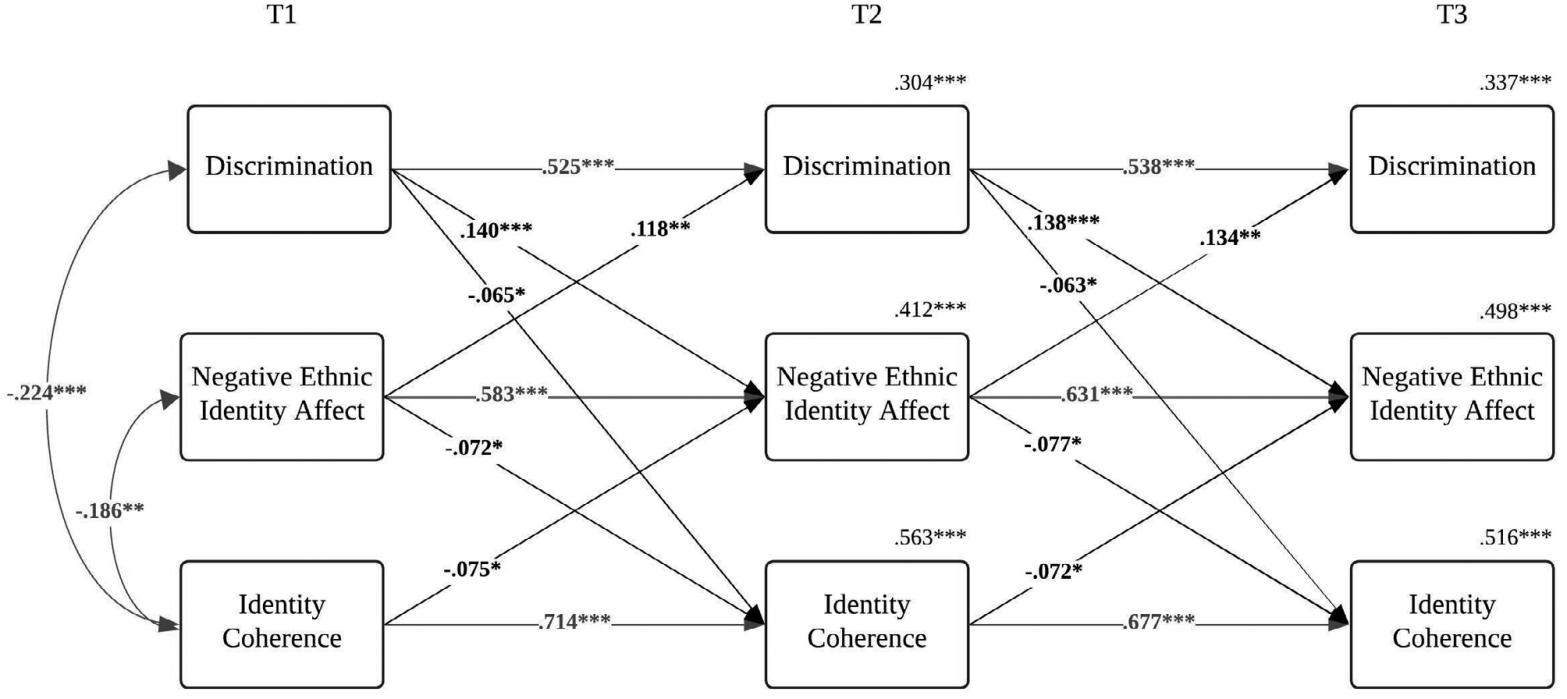
Standardized model results. For the sake of clarity, only significant standardized estimates are shown. The effects of adolescents' age and gender were accounted for. T, time. *p < .05; **p < .01; ***p < .001.

In addition to these significant cross‐lagged associations, all stability paths were positive and significant. However, a few significant within‐time correlations appeared only at T1. Specifically, identity coherence was negatively correlated with negative ethnic identity affect (*r* = −.186, *p* < .01) and negatively correlated with peer discrimination (*r* = −.224, *p* < .001). With regard to covariates' effects, males reported higher negative ethnic identity affect than females (*β* = −.071, *p* < .05 between T1 and T2; *β* = −.070, *p* < .05 between T1 and T3). Participants' age did not significantly affect any of the main study variables.

### Sensitivity analysis

Two sets of ancillary sensitivity analyses were conducted. First, the potential moderating roles of adolescents' participation in the (Identity Project) intervention and their generational status were examined separately. Regarding the intervention effects, multigroup analysis documented that the constrained model did not differ from the unconstrained model (${\Delta \chi }_{\mathrm{SB}}^{2}$ = 18.571, *df* = 18, *p* = .419, ΔCFI = −.003, and ΔRMSEA = −.006). Likewise, models did not differ across generational statuses (${\Delta \chi }_{\mathrm{SB}}^{2}$ = 13.199, *df* = 18, *p* = .780, ΔCFI = .006, and ΔRMSEA = −.011). Thus, all findings were consistent across intervention and control groups, as well as first‐generation and second‐generation ethnic minority adolescents.

Second, because of the nested structure of the data, the models were also (re)estimated using the *type = complex* function. As detailed in Supporting Information (Tables S5 and S6), the previously significant cross‐lagged paths from peer discrimination to identity coherence (*β* = −.065, *p* = .073 from T1 to T2; *β* = −.063, *p* = .075 from T2 to T3) and from negative ethnic identity affect to identity coherence (*β* = −.072, *p* = .068 from T1 to T2; *β* = −.077, *p* = .065 from T2 to T3) became nonsignificant. Nevertheless, given that the model with *type = complex* function produced warnings about a non‐positive definite product matrix, the findings of the initial model (with *type = general* function) were deemed the final one.

## DISCUSSION

Research to date has examined either the interplay between ethnic identity and global identity coherence (e.g., Meca et al., 2017) or the unique effects of discrimination on each (Meca, Webb, et al., 2022; Zeiders et al., 2019), while placing relatively limited emphasis on domain‐specific peer discrimination and its effects on both identities (see the exceptions for ethnic identity; Del Toro et al., 2021; Stein et al., 2023). The current study addresses this gap by disentangling the longitudinal interplay of peer discrimination with negative ethnic identity affect and identity coherence among ethnic minority youth. The main contributions of the study are threefold. First, it captures the bidirectional negative relationship between negative ethnic identity affect and global identity coherence. Second, it disentangles the bidirectional positive associations between peer discrimination and negative ethnic identity affect among ethnic minority youth. Finally, it reveals the detrimental impact of peer discrimination on identity coherence. The first and the last contributions largely expand the existing knowledge beyond the positive ethnic identity affect (i.e., affirmation) to negative feelings about one's ethnic group. The second contribution provides novel insights into the dynamic interplay between discrimination and negative ethnic identity affect among ethnic minority youth. Taken together, these contributions further speak to a certain level of relevance of U.S.‐based theories and findings for understanding the identity experiences of ethnic minority youth in the relatively understudied Italian cultural context.

### Negative bidirectional associations between negative ethnic identity affect and identity coherence

As expected, the current findings show that the links between negative ethnic identity affect and identity coherence are negative and bidirectional. While the current findings are somewhat inconsistent with the findings from Europe demonstrating that the personal and cultural dimensions of adolescents' identities were concurrently linked and co‐developed during adolescence (Mastrotheodoros et al., 2021), they extend the prior studies in which similar bidirectionality was observed for positive ethnic identity affect in the United States (Meca et al., 2017). Hence, the established bidirectional pattern within the current research may nonetheless indicate that the consolidation of both cultural and personal dimensions of the self might mutually be fostered.

On the one hand, adolescents who feel negatively about their sense of belonging to their ethnic and cultural group may find it harder to integrate this feeling into a coherent overall sense of self. On the other hand, forming a coherent sense of self may facilitate their ethnic identity because knowing “Who am I”, at least to some extent, may provide ethnic minority youth with the security and stability needed to feel less negative about their ethnic and cultural group memberships. Taken together, the observed mutual dynamics not only contribute to long‐standing discussions on how ethnic identity (as a cultural identity facet) develops vis‐à‐vis personal identity (Schwartz et al., 2006, 2008) by considering relatively understudied negative ethnic identity affect but also highlight the need for culturally sensitive psychosocial interventions that address how to overcome negative feelings towards one's ethnic group and its antecedents. Considering that identity interventions have been found effective for each identity facet per se (for a meta‐analysis, see Crocetti et al., 2025), they might likely be effective in promoting positive ethnic identity affect and a coherent sense of overall self by preventing negative feelings towards one's ethnic identity and breaking its negative feedback loop with identity coherence.

### Positive bidirectional associations between peer discrimination and negative ethnic identity affect

This study also identifies novel positive bidirectional links between peer discrimination and negative ethnic identity affect among ethnic minority youth in Italy. Specifically, when adolescents experienced greater peer discrimination at one time point, they later reported relatively higher negative feelings derived from their ethnic group membership. Conversely, when they endorsed higher negative feelings towards their ethnic group membership at one time point, they indicated higher levels of peer discrimination over time. This observed reciprocal pattern is somewhat inconsistent with conceptual models that imply negative associations between discrimination and negative ethnic identity affect due to increased awareness of ethnic group membership fostered by discrimination (i.e., RIM, Branscombe et al., 1999) or greater awareness and sensitivity of discrimination when they develop ethnic identity (IAM, Gonzales‐Backen et al., 2018). Instead, the current findings appear to align with the extant knowledge about the detrimental effects of peer discrimination on positive ethnic identity affect (see Del Toro et al., 2021; Stein et al., 2023) while also expanding it to negative ethnic identity affect.

The positive longitudinal associations between peer discrimination and adolescents' negative ethnic identity affect may be interpreted in light of the risk and resilience framework (Suárez‐Orozco et al., 2018). Exposure to peer discrimination may create a divisive social environment for youth's psychosocial adjustment through exacerbating negative feelings towards their ethnic group membership, inevitably weakening ethnic identity as an acculturative task. In fact, exposure to discrimination in peer groups may signal to ethnic minority youth that their ethnic and cultural backgrounds are devalued or even rejected. This may pose a high risk for forming strong and positive affective ties with their ethnic and cultural group while simultaneously increasing negative affective ties, which may in turn be detrimental to their psychosocial adjustment. Although such indirect associations have been demonstrated for positive ethnic identity affect in an adult sample (see Watkins et al., 2025), the interplay between peer discrimination and adjustment through both positive and negative ethnic identity affect during adolescence may be an important direction for future lines of inquiry.

Alternatively, the undesirable impacts of peer discrimination in exacerbating negative ethnic identity affect might be read in light of the broader sociocultural context, where many ethnic majority peers expect their ethnic minority counterparts to assimilate the majority Italian culture (Mancini & Bottura, 2014). This rather assimilative context might increase the risk that ethnic minority youth are exposed to discrimination in peer groups more often or intensify their sensitivity to peer discrimination. As a result, ethnic minority adolescents may hold less positive and even more negative feelings towards their own ethnic and cultural group. Indeed, a study involving ethnic minority youth in Italy with a comparable age range to those in the present sample found a significant decline in ethnic identification over time (Karataş, Crocetti, et al., 2023). Altogether, disentangling the role of perceived peer discrimination in driving the developmental trajectories of both positive and negative ethnic identity affect nonetheless awaits future investigation, particularly in cultural contexts where assimilative expectations are strongly endorsed.

The findings also indicate that negative ethnic identity affect is positively associated with peer discrimination over time. These findings may be interpreted in light of the possibility that negative feelings towards one's own ethnic identity might make young people more vulnerable to peer discrimination. An essential way to diminish youth's potential vulnerability derived from negative ethnic identity affect may be encouraging young people to develop “proactive coping” strategies. These strategies encompass talking directly to the person to clarify the misconception about ethnic groups (i.e., discussion), being proud of themselves and focusing on positive (self)attributes (i.e., self‐affirmation), and working hard to prove the person wrong (i.e., disapproval; see Umaña‐Taylor et al., 2008). Considering the negative correlations between positive and negative ethnic identity affect (Meca, Gonzales‐Backen, et al., 2022), their links with discrimination may also operate in opposite directions. Hence, fostering proactive coping strategies against discrimination and its detrimental effects on youth's mental health (e.g., Huynh & Fuligni, 2010; Schmitt et al., 2014), might be protective, most likely through making youth's feelings about ethnic identity more positive and less negative, which is often beneficial for mental health (see Rivas‐Drake et al., 2014).

In this regard, tailoring school‐based interventions that encourage ethnic minority youth to learn about how to proactively cope with such adverse experiences while also training their majority peers to intervene when discrimination occurs might be particularly beneficial, especially when considering its favorable impacts on youth's mental health (e.g., McDermott et al., 2019; Salcido & Stein, 2024). Although the Identity Project Intervention, from which the data for the current study were drawn, indirectly addresses certain elements of proactive coping strategies, tailoring a more direct form of intervention that combines all three components of proactive coping against peer discrimination might be warranted. Herein, self‐affirmation writing interventions addressing discrimination and proactive coping across multiple sessions might constitute a good example. Despite its differential focus, such an intervention conducted with adolescents in Germany was indeed found to be effective in the short‐term, particularly for those who experience higher discrimination, preventing a downward trajectory of being persistent in academic challenges (Juang et al., 2024).

As a secondary benefit, delivering school‐based interventions may also promote positive intergroup interactions among peers that are free from discrimination. Because both ethnic minority and majority youth tend to transfer their positive contact with outgroup members from one context to another (Karataş, Eckstein, et al., 2023), involvement in such interventions may ultimately equip them to apply their proactive coping skills to foster harmonious interactions within and beyond peer settings by impairing discrimination in itself and its undesirable consequences.

### Peer discrimination undermines global identity coherence in adolescence

Unlike the bidirectional patterns, unidirectional associations are found in which peer discrimination reduced identity coherence over time. This rather expected relational pattern complements prior findings (e.g., Meca, Webb, et al., 2022) emphasizing discrimination as a form of cultural stress that might hinder the formation of a coherent self. Along this line of thought, the first possible explanation might be that confronting peer discrimination in adolescence, during which peers (or friendships) become one of the central interpersonal domains of youth's identity formation (Crocetti, 2017), can exacerbate the impacts of stress on identity coherence. An alternative interpretation might be linked to how peer discrimination reduces emotional commitments to peer groups because identity formation processes are inextricably intertwined with emotional commitments to peer groups (i.e., classmates and group of friends; Albarello et al., 2018). Together, unrevealing the extent to which peer discrimination affects cultural stress and emotional commitments may provide an avenue for future research to better understand the mechanisms underlying the interplay between discrimination and identity coherence.

### Limitations, future directions, and concluding remarks

The current study is not without limitations. First, this longitudinal study adopts a domain‐specific approach and focuses on peer discrimination, yet the ethnic and cultural composition of peer groups was not accounted for (for example, see Crocetti et al., 2024). Ethnic minority youth may encounter discrimination beyond peers, such as teachers (Civitillo et al., 2024; Karataş et al., 2026) and non‐school adults (Del Toro et al., 2021). Future longitudinal research could therefore examine the unique and interactive effects of discrimination from different sources, while controlling for the ethnic composition of each.

Second, the present research captures ethnic identity development through assessing the extent to which youth feel negative about their ethnic and cultural group membership. In doing so, negatively worded items were utilized, unlike the long‐established practice of using reverse coding to assess positive ethnic identity affect (Meca, Gonzales‐Backen, et al., 2022). However, ethnic identity affect may consist of positive and negative feelings, both of which might be differently associated with self‐evaluation and adjustment outcomes (Umaña‐Taylor, 2024). Therefore, to more accurately reflect ethnic identity affect throughout adolescence, future longitudinal studies might distinguish and involve positive and negative feelings stemming from ethnic group membership.

Third, ethnic identity development is a multifaceted construct that involves both content and process dimensions. Similarly, the formation of coherent identities might also be examined through personal identity processes (i.e., commitment, in‐depth exploration, and reconsideration of commitment; Crocetti, 2017). Taken together, an integrative approach incorporating all process dimensions related to ethnic and personal identities (for example, see Cheon & Yip, 2019) might provide a refined approach to better understand the role of peer ethnic discrimination.

Fourth, the ethnic minority adolescents in this study were involved in the Identity Project intervention. Although the findings were fully replicated across intervention and control groups, the use of intervention data may still warrant consideration. Relatedly, the current study spanned only 13 weeks and can therefore be considered a short‐term longitudinal study with irregular intervals. As such, interpretation of the associations, especially those indicating bidirectionality, should be considered in the context of fairly high levels of stability in such a short time period. Conducting further longitudinal research with longer and regular time intervals and with a sample free of intervention conditions could provide a nuanced understanding of the dynamic interplay under examination.

Fifth, it is worth noting that this study followed the birth country‐based definition to determine youth's ethnic minority (versus majority) membership (European Commission, 2020). According to this classification, ethnic minority status somewhat overlaps with immigration status, although these constructs are conceptually distinct and warrant further attention, especially in exploring the discrimination‐identity link (e.g., Cheon & Yip, 2019). Given that findings in our research remained consistent across generational subgroups (for a similar pattern, see also Meca et al., 2020), alternative approaches to better capture the potential heterogeneity might still be considered. As a proxy for the determination of ethnic minority status, future research should incorporate psychometrically sound measures assessing adolescents' own perceptions of being viewed as foreigners regardless of their immigration status, citizenship, or self‐identification (i.e., foreigner objectification; Juang et al., 2021).

In spite of these and other limitations, the current research indicates the negative bidirectional associations between negative ethnic identity affect and global identity coherence among ethnic minority adolescents in Italy. Importantly, the findings also reaffirm the associations of peer discrimination with higher negative ethnic identity affect and lower global identity coherence, while reflecting that holding negative feelings about one's ethnic group may heighten the risk of being more sensitive and vulnerable to discrimination within peer contexts. Altogether, these findings offer a nuanced, domain‐specific perspective on peer discrimination and its longitudinal interplay with youth identity development within a relatively underrepresented sociocultural context of Italy. It is hoped that this research will inspire further domain‐specific and longitudinal work in this direction.

## AUTHOR CONTRIBUTIONS

**Savaş Karataş:** Conceptualization; investigation; funding acquisition; writing – original draft; methodology; formal analysis; data curation; project administration; writing – review and editing; software; resources; visualization. **Jana Vietze:** Conceptualization; funding acquisition; writing – review and editing; writing – original draft; project administration. **Chiara Ceccon:** Writing – original draft; writing – review and editing; data curation; funding acquisition; project administration. **Ughetta Moscardino:** Writing – original draft; writing – review and editing; funding acquisition; project administration; data curation.

## CONFLICT OF INTEREST STATEMENT

None of the authors have any conflicts of interest to disclose.

## ETHICS STATEMENT

All procedures performed in this study involving human participants were in accordance with the ethical standards of the Ethics Committee of the University of Padua (Protocol no 3871) (Italy).

## PARTICIPANT CONSENT STATEMENT

Informed consent was obtained from all individual participants (and from their parents, if minors) included in the study.

## Supporting information


**Table S1.** Participants' and Parents' National Origin.
**Table S2.** Longitudinal Measurement Invariance Tests Conducted with the Original Structures of the Study Measures.
**Table S3.** Standardized Factor Loadings Resulting from Longitudinal Measurement Invariance Tests for Each Study Measure.
**Table S4.** Longitudinal Measurement Invariance Tests for Each Study Measure After Excluding Poorly Loaded Items.
**Table S5.** Cross‐Lagged Models with TYPE = COMPLEX Function: Model Fit Indices and Model Comparisons.
**Table S6.** Standardized Results of the Cross‐Lagged Model with TYPE = COMPLEX Function.

## Data Availability

The data that support the findings of this study are available from the corresponding author upon reasonable request.
