# Supplementary material for: The costs of peer ethnic discrimination: Longitudinal associations with negative ethnic identity affect and identity coherence among ethnic minority adolescents
Source: J Res Adolesc. 2026 Sep 28;36(4):e70265. doi: 10.1111/jora.70265 (PMC13620443; doi:10.1111/jora.70265)
Supplement: Supplementary file 1 [file JORA-36-0-s001.docx]

**Supplementary Information for**

# “The Costs of Peer Ethnic Discrimination: Longitudinal Associations with Negative Ethnic Identity Affect and Identity Coherence among Ethnic Minority Adolescents”

**Table S1**

*Participants’ and Parents’ National Origin*

|  | *n* | % |
| --- | --- | --- |
| ***Participants’ country of birth*** |  |  |
| Albania | 1 | 0.36 |
| Bangladesh | 2 | 0.71 |
| Brazil | 2 | 0.71 |
| Burkina Faso | 1 | 0.36 |
| Cambodia | 1 | 0.36 |
| Cameroon | 1 | 0.36 |
| Colombia | 2 | 0.71 |
| Dominican Republic | 2 | 0.71 |
| Ethiopia | 1 | 0.36 |
| France | 1 | 0.36 |
| Germany | 1 | 0.36 |
| India | 1 | 0.36 |
| Italy | 214 | 76.43 |
| Mauritania | 1 | 0.36 |
| Moldova | 10 | 3.57 |
| Morocco | 8 | 2.86 |
| Nigeria | 1 | 0.36 |
| Pakistan | 3 | 1.07 |
| Philippines | 3 | 1.07 |
| Romania | 10 | 3.57 |
| Russia | 1 | 0.36 |
| Sri Lanka | 1 | 0.36 |
| Togo | 1 | 0.36 |
| Tunisia | 1 | 0.36 |
| Ukraine | 2 | 0.71 |
| Venezuela | 1 | 0.36 |
| Missing data | 7 | 2.50 |
| ***Parents’ country of birth*** |  |  |
| Albania | 39 | 6.96 |
| Azerbaijan | 1 | 0.18 |
| Australia | 1 | 0.18 |
| Bangladesh | 12 | 2.14 |
| Belarus | 1 | 0.18 |
| Belgium | 1 | 0.18 |
| Brazil | 8 | 1.43 |
| Burkina Faso | 2 | 0.36 |
| Cameroon | 8 | 1.43 |
| China | 41 | 7.32 |
| Colombia | 4 | 0.71 |
| Congo | 3 | 0.54 |
| Croatia | 1 | 0.18 |
| Cuba | 2 | 0.36 |
| Dominican Republic | 5 | 0.89 |
| Ecuador | 4 | 0.71 |
| Egypt | 2 | 0.36 |
| Ethiopia | 2 | 0.36 |
| France | 5 | 0.89 |
| Gambia | 2 | 0.36 |
| Germany | 2 | 0.36 |
| Guatemala | 1 | 0.18 |
| Hungary | 2 | 0.36 |
| India | 2 | 0.36 |
| Italy | 65 | 11.61 |
| Ivory Coast | 3 | 0.54 |
| Kosovo | 2 | 0.36 |
| Macedonia | 4 | 0.71 |
| Mauritania | 2 | 0.36 |
| Mexico | 1 | 0.18 |
| Moldova | 42 | 7.50 |
| Montenegro | 1 | 0.18 |
| Morocco | 71 | 12.68 |
| Netherlands | 2 | 0.36 |
| Nigeria | 30 | 5.36 |
| Pakistan | 6 | 1.07 |
| Philippines | 25 | 4.46 |
| Poland | 2 | 0.36 |
| Romania | 96 | 17.14 |
| Russia | 3 | 0.54 |
| Senegal | 1 | 0.18 |
| Serbia | 4 | 0.71 |
| Sierra Leone | 1 | 0.18 |
| South Korea | 1 | 0.18 |
| Spain | 2 | 0.36 |
| Sri Lanka | 2 | 0.36 |
| Sudan | 1 | 0.18 |
| Switzerland | 2 | 0.36 |
| Togo | 1 | 0.18 |
| Tunisia | 10 | 1.79 |
| Ukraine | 2 | 0.36 |
| USA | 1 | 0.18 |
| Venezuela | 2 | 0.36 |
| Missing data | 24 | 4.29 |

*Note*. Participants’ country of birth: *N* = 280. Parents’ country of birth: *N* = 560.

**Longitudinal Measurement Invariance Tests**

Before testing the Cross-Lagged Panel Model, we examined the longitudinal measurement invariance (van de Schoot et al., 2012) separately for the measures of interest: peer discrimination, negative ethnic identity affect, and identity coherence. The findings are displayed in Table S2. As functioning as the baseline, a configural model (M1) could be established for each study measure. The full metric invariance was achieved for the measures of peer discrimination and identity coherence, while partial metric invariance was reached for the measure of negative ethnic identity affect. Although the fit indices and model comparison results indicated metric invariance, standardized factor loadings of some items of the peer discrimination and identity coherence measures were relatively low (i.e., Items 4, see Table S3). Consequently, another set of longitudinal measurement invariance tests was conducted by excluding those poorly loaded items.

As displayed in Table S4, the findings of the second set of tests confirmed the presence of configural and full metric invariance for peer discrimination and identity coherence measures after excluding poorly loaded items. Given that all items were sufficiently loaded into one factor and the partial metric invariance was reached for the original structure of the negative ethnic identity affect measure, no secondary measurement invariance tests were necessary. In light of these findings, it is possible to proceed with analyses to unravel the longitudinal interplay of peer discrimination, negative ethnic identity affect, and identity coherence among ethnic minority youth.

**Table S2**

*Longitudinal Measurement Invariance Tests Conducted with the Original Structures of the Study Measures*

|  | **Model fit indices** | | | | | | **Model comparison** | | | | | |
| --- | --- | --- | --- | --- | --- | --- | --- | --- | --- | --- | --- | --- |
|  | *χ*_SB_^2^ | *df* | CFI | TLI | SRMR | RMSEA [90% CI] | Models | Δχ_SB_^2^ | Δ*df* | *p* | ΔCFI | ΔRMSEA |
| Peer Discrimination | | | | | | |  |  |  |  |  |  |
| M1. Configural model | 105.579 | 72 | .969 | .955 | .048 | .041 [.022, .057] |  |  |  |  |  |  |
| M2. Metric model | 116.069 | 80 | .967 | .956 | .053 | .040 [.023, .056] | M2-M1 | 10.238 | 8 | .249 | -.002 | -.001 |
| Negative Ethnic Identity Affect | | | | | | |  |  |  |  |  |  |
| M1. Configural model | 220.278 | 111 | .919 | .889 | .055 | .060 [.048, .071] |  |  |  |  |  |  |
| M2. Metric model | 244.232 | 121 | .909 | .885 | .065 | .061 [.050, .071] | M2-M1 | 24.084 | 10 | .007 | -.010 | .001 |
| M2a. Partial metric model | 231.707 | 119 | .917 | .893 | .062 | .058 [.047, .070] | M2a-M1 | 11.345 | 8 | .183 | -.002 | -.002 |
| Global Identity Coherence | | | | | | |  |  |  |  |  |  |
| M1. Configural model | 180.910 | 114 | .956 | .940 | .052 | .046 [.033, .058] |  |  |  |  |  |  |
| M2. Metric model | 192.224 | 124 | .955 | .944 | .056 | .045 [.032, .057] | M2-M1 | 10.512 | 10 | .397 | -.001 | -.001 |

*Note.* χ_SB_^2^ = Satorra-Bentler scaled chi-square; *df* = degrees of freedom; CFI = Comparative Fit Index; TLI = Tucker-Lewis Index; SRMR = Standardized Root Mean Square Residual; RMSEA [90% CI] = Root Mean Square Error of Approximation and 90% Confidence Interval; Δ = Change in the parameter.

**Table S3**

*Standardized Factor Loadings Resulting from Longitudinal Measurement Invariance Tests for Each Study Measure*

| **Variables** | **Items** | **Original Structure** | | | | | | **Revised Structure** | | | | | |
| --- | --- | --- | --- | --- | --- | --- | --- | --- | --- | --- | --- | --- | --- |
|  |  | **Configural** | | | **(Partial) Metric** | | | **Configural** | | | **(Partial) Metric** | | |
|  |  | **T1** | **T2** | **T3** | **T1** | **T2** | **T3** | **T1** | **T2** | **T3** | **T1** | **T2** | **T3** |
| **Peer Ethnic Discrimination** | Item 1 | .674 | .657 | .661 | .655 | .644 | .686 | .671 | .635 | .648 | .648 | .628 | .674 |
|  | Item 2 | .780 | .695 | .774 | .733 | .725 | .793 | .777 | .702 | .772 | .736 | .726 | .792 |
|  | Item 3 | .533 | .563 | .610 | .555 | .567 | .583 | .531 | .549 | .599 | .552 | .556 | .571 |
|  | Item 4 | **.278** | **.361** | **.362** | **.340** | **.313** | **.333** | *Excluded* | | | | | |
|  | Item 5 | .736 | .810 | .846 | .765 | .800 | .832 | .742 | .830 | .860 | .780 | .811 | .842 |
| **Negative** **Ethnic Identity Affect** | Item 1 | .516 | .710 | .593 | .524 | .712 | .609 |  | | | | | |
|  | Item 2 | .641 | .716 | .759 | .693 | .727 | .690 |  |  |  |  |  |  |
|  | Item 3 | .762 | .815 | .764 | .748 | .820 | .774 |  |  |  |  |  |  |
|  | Item 4 | .457 | .559 | .444 | .432 | .530 | .486 |  |  |  |  |  |  |
|  | Item 5 | .752 | .649 | .839 | .740 | .654 | .845 |  |  |  |  |  |  |
|  | Item 6 | .673 | .775 | .730 | .644 | .759 | .750 |  |  |  |  |  |  |
| **Identity Coherence** | Item 1 | .737 | .702 | .708 | .700 | .717 | .728 | .743 | .713 | .711 | .709 | .722 | .731 |
|  | Item 2 | .650 | .656 | .662 | .644 | .650 | .657 | .645 | .645 | .644 | .642 | .641 | .645 |
|  | Item 3 | .687 | .645 | .621 | .689 | .645 | .636 | .693 | .658 | .629 | .698 | .650 | .639 |
|  | Item 4 | .738 | .760 | .792 | .735 | .766 | .792 | .741 | .761 | .804 | .743 | .767 | .795 |
|  | Item 5 | **.198** | **.326** | **.311** | **.280** | **.278** | **.245** | *Excluded* | | | | | |
|  | Item 6 | .558 | .656 | .672 | .609 | .640 | .642 | .555 | .650 | .655 | .606 | .632 | .629 |

*Note.* Items with poor standardized factor loadings are shown in bold.

**Table S4**

*Longitudinal Measurement Invariance Tests for Each Study Measure After Excluding Poorly Loaded Items*

|  | **Model fit indices** | | | | | | **Model comparison** | | | | | |
| --- | --- | --- | --- | --- | --- | --- | --- | --- | --- | --- | --- | --- |
|  | *χ*_SB_^2^ | *df* | CFI | TLI | SRMR | RMSEA [90% CI] | Models | Δχ_SB_^2^ | Δ*df* | *p* | ΔCFI | ΔRMSEA |
| Peer Discrimination | | | | | | |  |  |  |  |  |  |
| M1. Configural model | 54.131 | 39 | .983 | .972 | .039 | .037 [.000, .060] |  |  |  |  |  |  |
| M2. Metric model | 61.752 | 45 | .981 | .973 | .044 | .037 [.002, .058] | M2-M1 | 7.433 | 6 | .283 | -.002 | .000 |
| Negative Ethnic Identity Affect | | | | | | |  |  |  |  |  |  |
| M1. Configural model | 220.278 | 111 | .919 | .889 | .055 | .060 [.048, .071] |  |  |  |  |  |  |
| M2. Metric model | 244.232 | 121 | .909 | .885 | .065 | .061 [.050, .071] | M2-M1 | 24.084 | 10 | .007 | -.010 | .001 |
| M2a. Partial metric model | 231.707 | 119 | .917 | .893 | .062 | .058 [.047, .070] | M2a-M1 | 11.345 | 8 | .183 | -.002 | -.002 |
| Global Identity Coherence | | | | | | |  |  |  |  |  |  |
| M1. Configural model | 115.133 | 72 | .969 | .954 | .045 | .047 [.030, .062] |  |  |  |  |  |  |
| M2. Metric model | 121.956 | 80 | .969 | .960 | .047 | .044 [.027, .058] | M2-M1 | 5.856 | 8 | .663 | .000 | -.003 |

*Note.* χ_SB_^2^ = Satorra-Bentler scaled chi-square; *df* = degrees of freedom; CFI = Comparative Fit Index; TLI = Tucker-Lewis Index; SRMR = Standardized Root Mean Square Residual; RMSEA [90% CI] = Root Mean Square Error of Approximation and 90% Confidence Interval; Δ = Change in the parameter.

**Table S5**

*Cross-Lagged Models with TYPE=COMPLEX Function: Model Fit Indices and Model Comparisons*

|  | **Model fit indices** | | | | |  | **Model comparison** | | | | |
| --- | --- | --- | --- | --- | --- | --- | --- | --- | --- | --- | --- |
| Models | *χ*^2^ | *df* | CFI | SRMR | RMSEA [90% CI] | Models | Δχ_SB_^2^ | Δ*df* | *p* | ΔCFI | ΔRMSEA |
| M1: Baseline model | 42.224 | 15 | .955 | .056 | .082 [.053, .111] |  |  |  |  |  |  |
| M2: Model with time-invariance of stability paths | 45.860 | 18 | .954 | .060 | .075 [.049, .103] | M2-M1 | 2.866 | 3 | .413 | -.001 | -.007 |
| M3: Model with time-invariance of stability paths and cross-lagged paths | 48.016 | 24 | .960 | .064 | .061 [.035, .086] | M3-M2 | 2.933 | 6 | .817 | .006 | -.014 |
| M4: Model with time-invariance of stability paths, cross-lagged paths, and T2– T3 correlations | 52.367 | 27 | .958 | .066 | .059 [.034, .082] | M4-M3 | 4.479 | 3 | .214 | -.002 | -.002 |
| M5: Model with time-invariance of stability paths, cross-lagged paths, and T2– T3 correlations, and covariate effects | 57.051 | 33 | .960 | .065 | .052 [.028, .074] | M5-M4 | 2.870 | 6 | .825 | .002 | -.007 |

*Note*. χ_SB_^2^ = Satorra-Bentler scaled chi-square; *df* = degrees of freedom; CFI = Comparative Fit Index; TLI = Tucker-Lewis Index; SRMR = Standardized Root Mean Square Residual; RMSEA [90% CI] = Root Mean Square Error of Approximation and 90% Confidence Interval; Δ = Change in the parameter.

**Table S6**

*Standardized Results of the Cross-Lagged Model with TYPE=COMPLEX Function*

| **Stability paths** | **T1 → T2** | **T2 → T3** |  |
| --- | --- | --- | --- |
| Peer discrimination | .525^***^ | .538^***^ |  |
| Negative ethnic identity affect | .583^***^ | .631^***^ |  |
| Identity coherence | .714^***^ | .677^***^ |  |
| **Cross-lagged paths** | **T1 → T2** | **T2 → T3** |  |
| Peer discrimination → Negative ethnic identity affect | .140^**^ | .138^***^ |  |
| Peer discrimination → Identity coherence | **-.065** | **-.063** |  |
| Negative ethnic identity affect → Peer discrimination | .118^***^ | .134^***^ |  |
| Negative ethnic identity affect → Identity coherence | **-.072** | **-.077** |  |
| Identity coherence → Peer discrimination | .006 | .006 |  |
| Identity coherence → Negative ethnic identity affect | -.075^*^ | -.072^*^ |  |
| **Covariate effects^1^** | **T1 → T2** | **T1 → T3** |  |
| Age → Peer discrimination | -.005 | -.005 |  |
| Age → Negative ethnic identity affect | -.024 | -.024 |  |
| Age → Identity coherence | -.011 | -.010 |  |
| Gender → Peer discrimination | .004 | .004 |  |
| Gender → Negative ethnic identity affect | -.071^*^ | -.070^*^ |  |
| Gender → Identity coherence | -.056 | -.054 |  |
| **Within-time correlations** | **T1** | **T2** | **T3** |
| Peer discrimination ↔ Negative ethnic identity affect | .126 | .030 | .033 |
| Peer discrimination ↔ Identity coherence | -.224^**^ | -.024 | -.023 |
| Negative ethnic identity affect ↔ Identity coherence | -.186^**^ | -.088 | -.086 |

*Note.* T = Time; ^*^ *p* < .05, ^**^ *p* < .01, ^***^ *p* < .001. Values that differ from the original findings (see Table 3) are displayed in bold.

**References**

van de Schoot, R., Lugtig, P., & Hox, J. (2012). A checklist for testing measurement invariance. *European Journal of Developmental Psychology, 9,* 486−492. <https://doi.org/10.1080/17405629.2012.686740>
